# Effect of nitrate supplementation on skeletal muscle motor unit activity during isometric blood flow restriction exercise

**DOI:** 10.1007/s00421-022-04946-y

**Published:** 2022-04-23

**Authors:** Ozcan Esen, Azmy Faisal, Fabio Zambolin, Stephen J. Bailey, Michael J. Callaghan

**Affiliations:** 1 Department of Health Professions, Manchester Metropolitan University, Manchester, M15 6GX UK; 2 Department of Sport and Exercise Sciences, Manchester Metropolitan University, Manchester, UK; 3 Manchester Metropolitan University Institute of Sport, Manchester, UK; 4 Faculty of Physical Education for Men, Alexandria University, Alexandria, Egypt; 5 School of Sport, Exercise and Health Sciences, Loughborough University, Loughborough, UK; 6 Manchester University Hospital Foundation Trust, Manchester, UK; 7 Arthritis Research UK Centre for Epidemiology, Centre for Musculoskeletal Research, Manchester Academic Health Science Centre, University of Manchester, Manchester, UK

**Keywords:** Nitric oxide, Beetroot juice, Electromyography, Muscle function, Neuromuscular function

## Abstract

**Background:**

Nitrate (NO_3_^−^) supplementation has been reported to lower motor unit (MU) firing rate (MUFR) during dynamic resistance exercise; however, its impact on MU activity during isometric and ischemic exercise is unknown.

**Purpose:**

To assess the effect of NO_3_^−^ supplementation on knee extensor MU activities during brief isometric contractions and a 3 min sustained contraction with blood flow restriction (BFR).

**Methods:**

Sixteen healthy active young adults (six females) completed two trials in a randomized, double-blind, crossover design. Trials were preceded by 5 days of either NO_3_^−^ (NIT) or placebo (PLA) supplementation. Intramuscular electromyography was used to determine the *M. vastus lateralis* MU potential (MUP) size, MUFR and near fibre (NF) jiggle (a measure of neuromuscular stability) during brief (20 s) isometric contractions at 25% maximal strength and throughout a 3 min sustained BFR isometric contraction.

**Results:**

Plasma nitrite (NO_2_^−^) concentration was elevated after NIT compared to PLA (475 ± 93 vs. 198 ± 46 nmol L^−1^, *p* < 0.001). While changes in MUP area, NF jiggle and MUFR were similar between NIT and PLA trials (all *p* > 0.05), MUP duration was shorter with NIT compared to PLA during brief isometric contractions and the sustained ischemic contraction (*p* < 0.01). In addition, mean MUP duration, MUP area and NF jiggle increased, and MUFR decreased over the 3 min sustained BFR isometric contraction for both conditions (all *p* < 0.05).

**Conclusions:**

These findings provide insight into the effect of NO_3_^−^ supplementation on MUP properties and reveal faster MUP duration after short-term NO_3_^−^ supplementation which may have positive implications for skeletal muscle contractile performance.

## Introduction

Dietary nitrate (NO_3_^−^) supplementation has been reported to increase plasma nitrite (NO_2_^−^) concentration, which can be reduced to nitric oxide (NO) and subsequently enhance skeletal muscle perfusion, metabolism and endurance performance (Jones et al. 2018). With regard to neuromuscular function, NO_3_^−^ supplementation has been reported to improve peak tetanic force in the quadriceps muscle during low-frequency (≤ 20 Hz) stimulation (Haider and Folland 2014). Subsequent studies reported that NO_3_^−^ supplementation could increase evoked quadriceps contractile force in fatigued, but not non-fatigued, muscle (Tillin et al. 2018), and with, but not without, lower limb blood flow restriction (BFR) (Hoon et al. 2015). In addition to inconsistent effects of NO_3_^−^ supplementation on neuromuscular function, its effect on motor unit (MU) activity, assessed using surface electromyography (sEMG) (Bailey et al. 2010; Haider and Folland 2014; Tillin et al. 2018; Husmann et al. 2019), particularly during voluntary contractions, is poorly understood having only been assessed in two previous studies (Flanagan et al. 2016; Porcelli et al. 2016). While Flanagan et al. (2016) reported a decrease in MU firing rate (MUFR) and an increase in sEMG peak amplitude after fatiguing dynamic box squat exercise, Porcelli et al. (2016) found no differences in root mean square sEMG values during fatiguing intermittent submaximal isometric knee extensions. However, sEMG might be limited due to the distance between activated MUs and recording electrodes and influenced by adjacent muscles. The use of a novel intramuscular electromyography (iEMG) technique in the present study can expand previous observations by overcoming limitations inherent in sEMG as well as revealing further electrophysiological parameters relevant to MU potential (MUP) size, stability and MUFR (Piasecki et al. 2021). Furthermore, Flanagan et al. (2016) administered a sport bar that provided a small NO_3_^−^ (~ 0.5 mmol/day) dose. As such, it is possible that other nutrients in the bar may have contributed to the effects observed. Evaluating the effect of NO_3_^−^ supplementation, at an appropriate NO_3_^−^ dose, on MU activity is important to improve understanding of the potential of NO_3_^−^ supplementations to modulate neuromuscular function.

A physiological increase in NO following dietary NO_3_^−^ has the potential to alter MU activity by modulating neurotransmitter release at the neuromuscular junction (NMJ). Indeed, it has been suggested that NO facilitates neurotransmitter release at the NMJ (Nickels et al. 2007; Zhu et al. 2013; Robinson et al. 2018) by two distinct mechanisms: (1) via activation of soluble guanylyl cyclase (sGC)—and the resultant increase in intracellular levels of cyclic guanosine monophosphate (cGMP), (2) s-nitrosylation of cysteine (Cys) thiol/sulfhydryl groups, on key regulatory proteins (Gould et al. 2013). In addition, NO_3_^−^ supplementation has been reported to lower plasma potassium (K^+^) concentration during exhaustive exercise, which may translate into preserved muscle excitability during fatigue-inducing contractions (Wylie et al. 2013b). However, while these data indirectly suggest that NO_3_^−^ supplementation has the potential to influence MU activity, empirical evidence to support this in humans is presently lacking.

Most previous studies that have investigated the effect of NO_3_^−^ supplementation on force production and neuromuscular function have implemented electrically stimulated muscle contractions (Haider and Folland 2014; Hoon et al. 2015; Tillin et al. 2018). These studies have attributed enhanced evoked contractile force to improved skeletal muscle calcium (Ca^2+^) handling, based on increased Ca^2+^ handling proteins, Ca^2+^ release and evoked contractile force in mouse fast-twitch, but not slow-twitch, muscle after NO_3_^−^ supplementation (Hernandez et al. 2012). However, translation of the data from the Hernandez et al. (2012) study in mouse muscle ex vivo to human muscle in vivo is complicated by the fact that the human quadriceps muscle is typically comprised of a heterogenous pool of muscle fibre types (Johnson et al. 1973; Anderson 2001) and that Ca^2+^ handling proteins are not increased in human skeletal muscle after NO_3_^−^ supplementation (Whitfield et al. 2017). Moreover, the translation of findings from involuntary contractions to voluntary contractions is confounded by disparate neuromuscular responses between these methods of muscle contraction (Bickel et al. 2011), with voluntary contractions fundamentally regulated by neuromuscular activation rather than the contractile properties of the muscle (Folland et al. 2014). Importantly, MUs are not necessarily recruited in order of size during stimulated involuntary contractions and their recruitment depends on proximity to the stimulating electrode which may lead to localised regions of fatigue (Jubeau et al. 2007). Conversely, during voluntary contractions, MUs are recruited in size order (small to large), and active MUs are typically spatially distributed through the muscle belly to minimise effects of localised fatigue (Henneman et al. 1965; Jubeau et al. 2007). As such, further research is required to assess the effect of NO_3_^−^ supplementation on neuromuscular function during voluntary contractions in humans.

Alterations in muscle force with NO_3_^−^ supplementation may also be linked to effects on MUFR and stability of NMJ transmissions that measured by jiggle [(a measure of the variability of MUPs amplitude across consecutive MU discharges (Allen et al. 2015)], particularly during fatiguing contractions, where BFR is present. While NO_3_^−^ supplementation has been shown to delay the development of fatigue (Hoon et al. 2015; Flanagan et al. 2016), and fatigue is associated with a reduction of MUFR (Carpentier et al. 2001; Enoka and Fuglevand 2001), it remains unknown whether NO_3_^−^ supplementation changes MUFR during prolonged and/or fatiguing contractions. Thus, the aim of this study was to investigate whether NO_3_^−^ supplementation alters MU activities during brief isometric knee extensor contractions and a 3 min sustained isometric contraction completed with BFR in young healthy adults. Contractions were completed with BFR in the current study, since BFR will lead to lower muscle PO_2_ and pH during contractions, conditions which would be expected to aid the reduction of NO_2_^−^ to NO (Modin et al. 2001; Castello et al. 2006), and since NO_3_^−^ supplementation appears more likely to improve neuromuscular function with BFR (Hoon et al. 2015). We hypothesized that (i) MUP size and jiggle would increase and MUFR would decrease during the sustained contraction with BFR; and (ii) NO_3_^−^ supplementation would blunt the increase in MUP size and jiggle, and the decrease in MUFR, during this contraction protocol.

## Methodology

### Participants

Sixteen healthy, physically active, non-smoking young adults (6 females) participated in this study (age 25 ± 6 years, body mass 71 ± 11 kg, height 1.74 ± 0.1 m; mean ± SD). Participants did not currently, or in the previous 3 months, have a musculoskeletal injury. All female participants in this study were using hormonal contraceptives. The study was conducted in accordance with the Declaration of Helsinki and approved by the Manchester Metropolitan University Research Ethics Committee (Approval ID: 5951). All participants provided written informed consent prior to participation in the study.

### Experimental design and procedures

Participants visited the laboratory to perform two experimental trials following 5 days of NO_3_^−^ (NIT) or placebo (PLA) supplementation following a randomized, double-blind, crossover design. A 7 ± 1 day washout period separated the supplementation periods (Wylie et al. 2013a). Experimental trials of each participant were scheduled at the same time of day (± 2 h). Participants were requested to maintain habitual physical activity, and to record their dietary intake in 3 days before the first experimental trial and to repeat the same diet during the 3 days prior to the subsequent visit. Participants were asked to arrive at the laboratory hydrated, to refrain from vigorous exercise, and not consume alcohol, caffeine and nutritional supplements 24 h before each trial visit, and not to use antibacterial mouthwash throughout the experimental period.

**Fig. 1:**
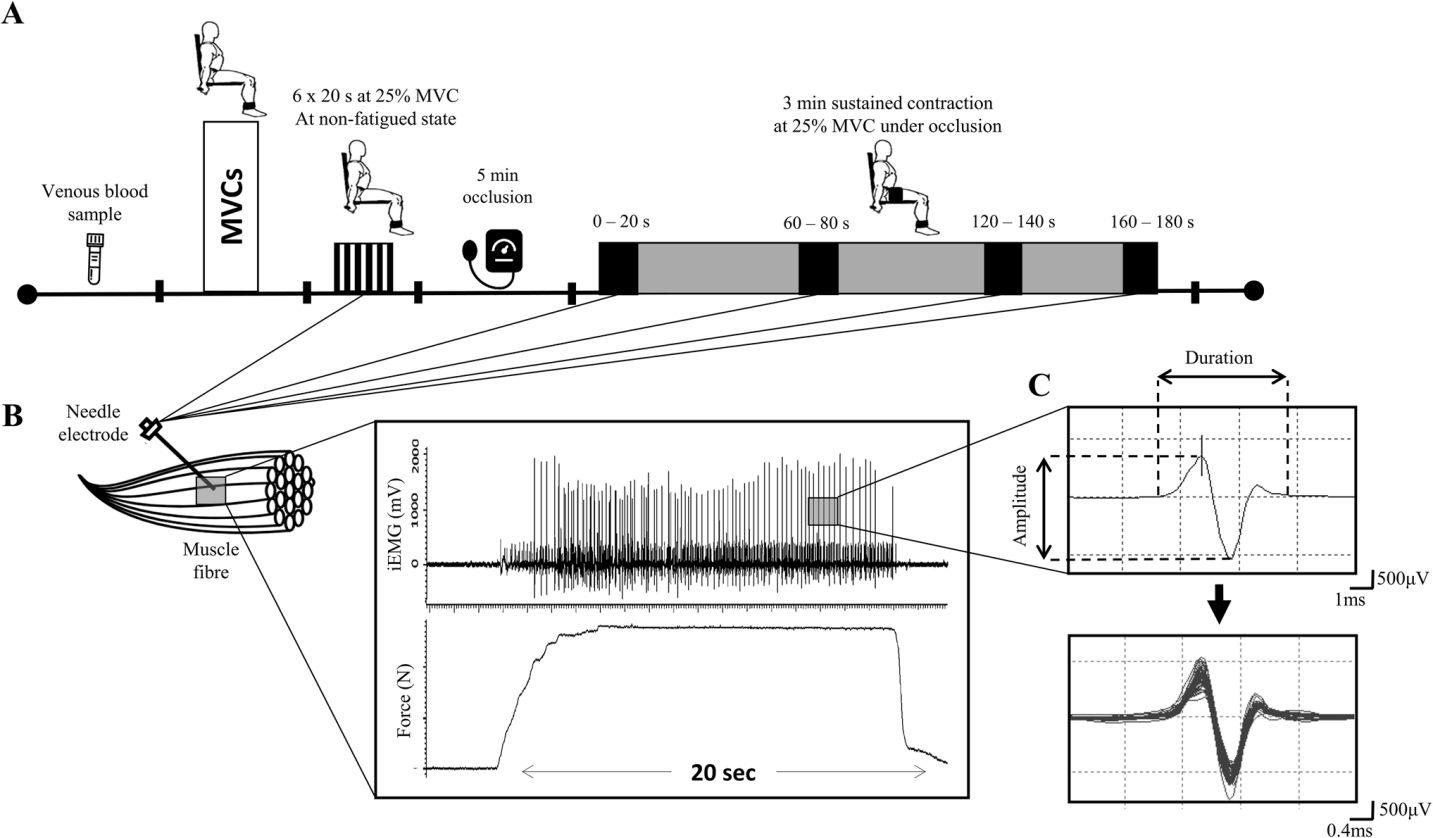
Experimental procedure and recorded force and intramuscular electromyography (iEMG) traces. **A** Schematic of the dynamometer, muscle contractions procedure and blood sample measurements. **B** Motor unit potential (MUP) overlay and force tracing from a representative participant during a 20 s isometric contraction at 25% maximal voluntary contrction (MVC). **C** Single MUP isolated from the traces shown in B and traces of the same MUP overlaid from consecutive firing, respectively

Each experimental trial required participants to complete the testing protocol (Fig. 1) with the dominant leg, determined as the preferred leg to kick a ball with. Participants first performed maximum voluntary contractions (MVCs) following a blood sample collection. Participants then performed brief submaximal isometric contractions, followed by 8 min of knee BFR period with a sustained submaximal contraction in the final 3 min of the BFR period. Ratings of perceived exertion (RPE) was recorded at the end of 3 min ischemic contraction using the Borg 6–20 scale (Borg 1998). iEMG was recorded from the *M. vastus lateralis* (VL) muscle during brief isometric contractions and throughout the 3 min sustained isometric contraction with BFR.

### Supplementation

Supplements were allocated in a double-blind design by an independent technician who did not take part in the assessments. Participants consumed 2 × 70 mL/day of concentrated NO_3_^−^-rich (NIT: ~ 12.8 mmol/day NO_3_^−^) or NO_3_^−^-depleted (PLA: ~ 0.08 mmol/day NO_3_^−^) beetroot juice (Beet It, James White Drinks Ltd., Ipswich, UK) for the two 5 days supplementation periods. Participants ingested two 70 mL shots per day for 4 days of supplementation: one each morning (~ 9 am) and one each evening (~ 9 pm). On the day of experimental trial, 2 × 70 mL shots were ingested together 2.5 h prior to the trial (Wylie et al. 2013a).

### Strength assessment

MVC of the knee extensors was assessed with the participant sitting with hip and knees flexed at 90°. The dominant leg was fastened into a custom-built dynamometer (Jones et al. 2009) 30 cm below the centre of the knee joint. The chest and waist were strapped to the chair to reduce extraneous movements. Participants performed a series of submaximal contractions for familiarization with the equipment and warm up. After completing a warm-up, participants completed 4 MVCs (~ 30 s apart), each lasting approximately 4 s with real-time visual feedback and verbal encouragement provided. The highest value was taken using a 200 ms moving average across the four data files and was used to represent MVC force.

### Intramuscular electromyography setup

iEMG signals were recorded by inserting a 25 mm disposable concentric needle electrode (Model S53156; Teca, Hawthorne,NY, USA) to a depth of 1–2 cm into the mid muscle belly of VL. A common ground electrode was placed over the patella. The iEMG signals were bandpass filtered at 10 Hz to 10 kHz and sampled at 40 kHz. iEMG signals and the force signal were recorded and displayed in real-time using LabChart8 software (v8.1.13, Adintstruments Products).

### Isometric contractions and motor units recording

The skin was prepared by shaving, lightly abrasing, and cleansing with 70% ethanol. Subsequently the needle position was adjusted to ensure that intramuscular MUPs were recorded (Stashuk 1999). Then, 6 × 20 s brief isometric contractions at 25% MVC with signal recording were completed with real-time visual feedback of the force each interspersed by ~ 30 s. The needle was repositioned via combinations of rotating 180° and withdrawing by 2–5 mm, respectively, after every contraction.

Following brief isometric contractions, a 13 cm wide cuff (Hokanson E20 cuff inflator; Bellevue, WA) was placed around the upper thigh of the right leg, just below the inguinal crease and inflated to 220 mmHg for 5 min to occlude arterial and venous lower leg blood flow (Mullen et al. 2011), a procedure demonstrated to expedite fatigue development compared to standard experimental conditions (Wernbom et al. 2006). The needle was then re-inserted into a new location at least 0.5 cm away from the original detection site, and participants completed a continuous 3 min ischemic contraction at 25% MVC with a stable needle position throughout. During the 3 min BFR protocol, iEMG sampling was recorded for 20 s at the start of the contraction (0–20 s), start of the second (60–80 s) and third minutes (120–140 s), and at the end of the third minute (160–180 s).

### Intramuscular electromyography analyses

**Fig. 2:**
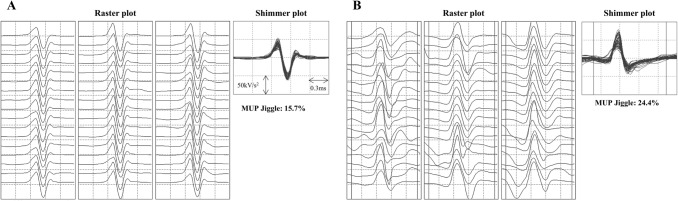
Motor unit potentials (MUPs) in brief isometric contraction (**A**) and during ischemic contraction (**B**). In each condition, 51 consecutive MUPs are shown in a raster plot and overlaid in respective shimmer plots. NF Jiggle tracks changes in consecutive MUP shapes and is expressed as a percentage of the total MUP area. Higher values indicate increased instability of neuromuscular transmission

iEMG signals were analysed via decomposition-based quantitative electromyography, as described elsewhere (Stashuk 1999; Piasecki et al. 2016a). Briefly, extracted MUP trains (MUPTs) with less than 40 MUPs were excluded (Fig. 2). All MUP templates were visually examined, and their markers (the onset, end, and positive and negative peaks of the waveforms) repositioned, where required, for accuracy. The MUP duration (ms) was measured between the onset and end of the waveforms. MUP area consisted of duration and peak-to-peak amplitude (μV ms).  Near fibre (NF) Jiggle represents the variability of consecutive MUP shape of MUPTs, and was expressed as a percentage of the total template MUP area (Hourigan et al. 2015). MUFR was determined from consecutive observations of the same MUP, expressed as number of observations per second (Hz) (Piasecki et al. 2016a, b).

### Plasma NO_2_^−^

Resting blood samples were collected to determine plasma NO_2_^−^ concentration on day 5 of both supplementation periods, at least 2.5 h after the last meal. A 5 mL venous blood sample was collected into a lithium–heparin tube (Vacutainer, Becton Dickinson) and immediately centrifuged at 1160×*g* and 4 °C for 10 min (hettich^®^ 320 centrifuge, Canada). Following centrifugation, plasma was extracted and frozen at − 80 °C for later analysis of [NO_2_^−^] using a modification of the chemiluminescence technique as previously described (Wylie et al. 2013b).

### Statistical analysis

A paired *t* test assessed differences between NIT and PLA supplements in plasma NO_2_^-^ and MVC, and MUP size, NF jiggle and MUFR in brief muscle contraction. Cohen’s *d* effect sizes were determined for each paired comparison (Cohen 1988). Two-way repeated measures ANOVAs were applied to assess for supplementation × time interactions for MUP size, NF jiggle and MUFR. If there was a significant main or interaction effect, Bonferroni corrected paired t tests were applied as post-hoc paired comparisons. All data were analysed using SPSS 26.0 (IBM Corp., Armonk, NY), and presented as mean ± SD. Absolute probability (*p* value) were reported except in cases, where *p* ≤ 0.001, and effect sizes were calculated as Partial eta square (*η*_*p*_^2^), which varies from moderate (≥ 0.07) to a large effect (≥ 0.14) (Cohen 1988).

## Results

Plasma [NO_2_^−^] concentration was higher in NIT than PLA (475 ± 93 nmol L^−1^ vs. 198 ± 46 nmol L^−1^, *p* < 0.001, *d* = 3.37). There was no significant difference in the MVC between NIT and PLA trials (984 ± 124 N vs. 945 ± 117 N; *p* = 0.243, *d* = 0.32). There was also no significant difference in RPE at the end of the 3 min sustained isometric contraction between NIT and PLA trials (18.0 ± 1.5 AU vs. 18.3 ± 1.5 AU; *p* = 0.703,  *d* = 0.2).

### Neuromuscular responses during isometric contractions

**Fig. 3:**
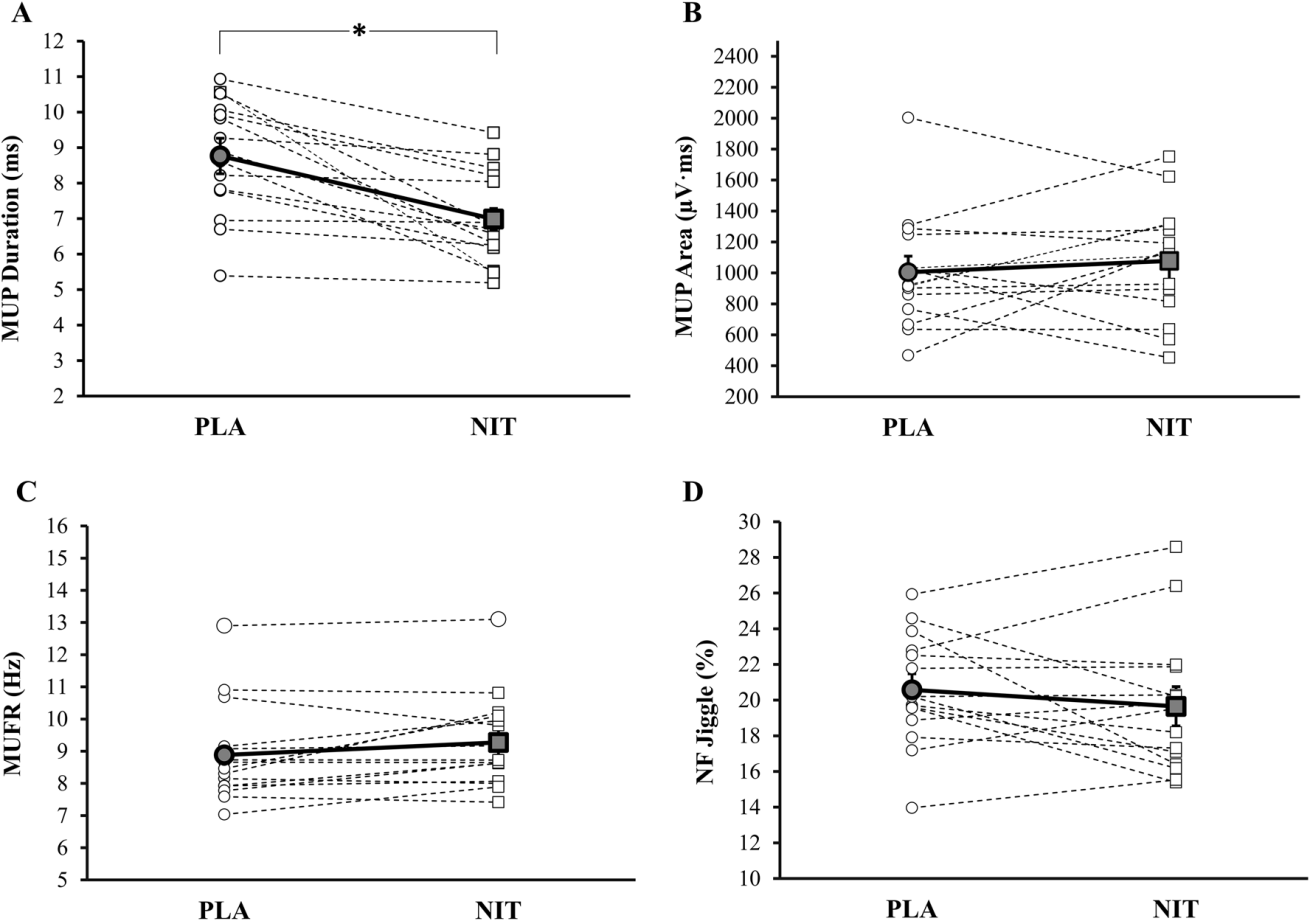
Motor unit potential (MUP) duration (**A**), MUP area (**B**), MU firing rate (MUFR, **C**) and NF jiggle (**D**) during contractions completed in brief isometric contraction after nitrate-rich (NIT) and nitrate-depelted (PLA) beetroot juice supplementaiton. Data are mean ± SD. *significance, *p* < 0.05

A mean number of MUs sampled per person during brief isometric contractions was 34 ± 7 for NIT and 33 ± 9 for PLA.The MUP duration was shorter in NIT than PLA (7.1 ± 0.3 ms vs. 9.0 ± 0.5 ms, *p* < 0.001, *d* = 4.61, Fig. 3A). There was no significant difference in the MUP area between NIT and PLA trials (1180.9 ± 129.0 µV ms vs. 1004.4 ± 104.6 µV ms, *p* = 0.283, *d* = 1.5, Fig. 3B). MUFR tended to be greater in NIT than PLA but did not reach statistically significant level (9.4 ± 0.4 Hz vs. 8.6 ± 0.3 Hz, *p* = 0.057, *d* = 2.26, Fig. 3C). There was also no significant difference in NF jiggle between NIT and PLA trials (19.8 ± 1.1% vs. 20.6 ± 0.9%, *p* = 0.320, *d* = 0.99, Fig. 3D).

### Neuromuscular responses during sustained isometric contractions with BFR

**Fig. 4:**
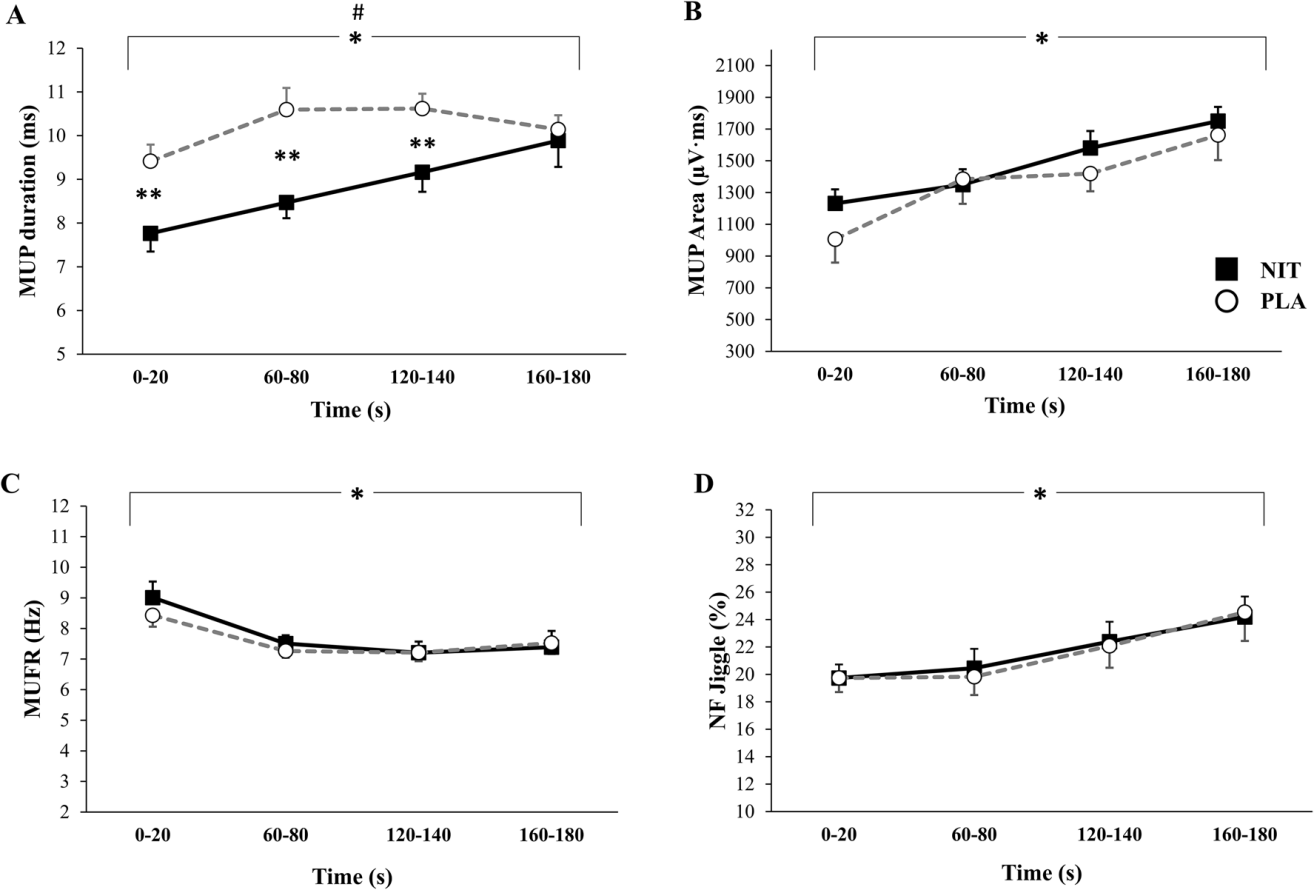
Motor unit potential (MUP) duration (**A**), MUP area (**B**), MU firing rate (MUFR, **C**) and NF jiggle (**D**) during a 3 min ischemic isometric contraction completed after nitrate-rich (NIT) and nitrate-depelted (PLA) beetroot juice supplementaiton. Data are mean ± SD. #Main effect of supplement *p* < 0.05. *Main effect of time, *p* < 0.05. **Post hoc comparisons at specific time points with Paired *t* test *p* < 0.05

A mean number of MUs sampled per person during 3 min ischemic contractions was 8 ± 2 vs. 8 ± 3 at 0–20 s; 9 ± 2 vs. 10 ± 3 at 60–80 s; 8 ± 1 vs. 9 ± 3 at 120–140 s; and 7 ± 3 vs. 8 ± 2 160–180 s for NIT and PLA, respectively. This number are slightly higher than the findings of the previous study which report reliability of this number during fatiguing contraction in arm muscle (Calder et al. 2008). MUP duration: There was a main effect of supplementation (*F* = 19.85; *p* = 0.001; *ŋ*_*p*_^2^ = 0.604), a main effect of time (*F* = 4.97; *p* = 0.025; *ŋ*_*p*_^2^ = 0.277) and supplementation × time interaction effect and on MUP duration (*F* = 5.23; *p* = 0.006; *ŋ*_*p*_^2^ = 0.287, Fig. 4A). Post-hoc paired comparisons showed that MUP duration was significantly shorter in NIT than PLA during the 0–20 s (7.8 ± 0.4 ms vs. 9.4 ± 0.4 ms, *p* < 0.001), 60–80 s (8.5 ± 0.4 ms vs. 10.6 ± 0.5 ms, *p* < 0.001) and 120–140 s (9.2 ± 0.4 ms vs. 10.6 ± 0.3 ms, *p* = 0.005) timepoints. MUP area: There was no significant supplementation × time interaction effect (*F* = 0.83; *p* = 0.488; *ŋ*_*p*_^2^ = 0.060) nor a main effect of supplementation on MUP area (*F* = 0.50; *p* = 0.492; *ŋ*_*p*_^2^ = 0.037, Fig. 4B). There was a main effect of time on MUP area (*F* = 17.24; *p* < 0.001; *ŋ*_*p*_^2^ = 0.57). Paired comparisons showed that MUP area was smaller at 0–20 s than 60–80 s (p = 0.001), 120–140 s (p = 0.001) and 160–180 s (p = 0.001); and at 60–80 s than 160–80 s in both conditions (*p* = 0.040). MUFR: There was no supplementation × time interaction effect (*F* = 0.30; *p* = 0.703; *ŋ*_*p*_^2^ = 0.027) nor a main effect for supplementation (*F* = 0.727; *p* = 0.412; *ŋ*_*p*_^2^ = 0.062, Fig. 4C) on MUFR. There was a main effect of time on MUFR (*F* = 6.458; *p* = 0.011; *ŋ*_*p*_^2^ = 0.370). Post-hoc paired comparisons showed that MUFR was higher at 0–20 s than 60–80 s (*p* = 0.003) and 120–140 s (*p* = 0.013). NF Jiggle: There was no significant supplementation × time interaction effect (*F* = 0.03; *p* = 0.994; *ŋ*_*p*_^2^ = 0.002) nor a main effect for supplementation on NF jiggle (*F* = 0.139; *p* = 0.716; *ŋ*_*p*_^2^ = 0.011, Fig. 4D). There was a significant main effect of time on NF jiggle (*F* = 3.87; *p* = 0.009; *ŋ*_*p*_^2^ = 0.260). Paired comparisons revealed that there was higher NF jiggle at 160–180 s than 0–20 s (*p* = 0.038).

## Discussion

A novel contribution of this study was the use of iEMG and decomposition-enhanced quantitative EMG to assess MU activities following dietary NO_3_^−^ supplementation during brief submaximal isometric contractions, and a sustained isometric contraction with BFR. The primary findings were that MUP area, NF jiggle and MUFR were similar in the NIT and PLA trials, but MUP duration was shorter in NIT compared to PLA during brief isometric contractions and a sustained contraction with BFR. In addition, mean MUP duration, MUP area and NF jiggle increased, and MUFR decreased, during the sustained isometric contraction conducted with BFR, indicating activation of larger MUs. Collectively, these findings reveal an increasing instability of neuromuscular transmissions during a sustained isometric, ischaemic contraction. The short-term high-dosage NO_3_^−^ supplementation regime reduced MUP duration, but had no effects on MUP area, NF jiggle and MUFR, during single isometric contractions and during a sustained ischemic isometric contraction in healthy active young adults. These findings improve understanding of the effects of NO_3_^−^ supplementation on neuromuscular function and which may have positive implications for skeletal muscle contractile performance.

### Plasma NO_2_^−^ concentration

Plasma NO_2_^−^ concentration increased after 5 day NO_3_^−^ supplementation and this increase was 140% higher compared to placebo, suggesting appreciably enhanced potential for NO synthesis through reduction of NO_2_^−^ to NO (Lundberg et al. 2008). This result is consistent with previous studies (e.g., Bailey et al. 2010; Wylie et al. 2013b; Tillin et al. 2018; Esen et al. 2019) and indicates the potential for NO synthesis to attenuate exercise-induced fatigue (Bailey et al. 2010; Tillin et al. 2018).

### MUP size

Increased MUP duration during an isometric contraction with BFR (Fig. 4A, B) is consistent with previous studies investigating the effect of fatigue on MUP duration during a sustained submaximal contraction (Calder et al. 2008; McManus et al. 2015). Increased MUP duration during fatigue development is accompanied by slowed muscle fiber conduction velocity (MFCV) (Calder et al. 2008; McManus et al. 2015; Mallette et al. 2021). Therefore, shorter MUP duration in the NIT condition might be linked to a faster action potential propagation along muscle fibres. Given that a reduction in muscle excitability, particularly during fatigue development, is partly related to a net loss of muscle K^+^ (McKenna et al. 2008), shorter MUP duration might be due to the potential for NO_3_^−^ supplementation to attenuate muscle K^+^ efflux and accumulation in the extracellular fluids (Wylie et al. 2013a). In addition, since there is some evidence that NO may facilitate neurotransmitter release at the NMJ via s-nitrosylation of cysteine thiols/sulfhydryl groups on key regulatory proteins (Nickels et al. 2007; Zhu et al. 2013; Robinson et al. 2018), shorter MUP duration following NO_3_^−^ supplementation might be linked to greater NO-induced acetylcholine release and subsequently faster MFCV (Rutkove, 2001). While muscle contractile force and the mechanisms for altered MUP were not assessed in the current study, shorter MUP duration may have resulted in a faster MFVC and greater sarcoplasmic reticulum Ca^2+^ release and force production (Murakami et al. 2014; Del Vecchio et al. 2018), or maintained force output in the face of fatigue development (Farina et al. 2005; McManus et al. 2015). Indeed, Ca^2+^ release from the sarcoplasmic reticulum is correlated to the speed of the action potential on the fiber membrane (Farina et al. 2005). However, further research is required to verify this postulate.

MUP area is a product of MUP duration and amplitude, but it is primarily determined by MUP amplitude (Calder et al. 2008; Piasecki et al. 2021). As such, a change in MUP area may occur independent of a change in MUP duration, because the MUP amplitude provides a third source of variation which in turn is influenced by motor unit size and the distance from the recording electrode (Piasecki et al. 2021). Indeed, increased MUP amplitude, and hence area, links to the recruitment of additional larger MUs instead of the ionic disturbances (Adam and De Luca 2005; Calder et al. 2008; McManus et al. 2015; Mallette et al. 2021; Guo et al. 2022) to compensate for the reduction in the force-generating capacity of the muscle (Bigland-Ritchie et al. 1986; Carpentier et al. 2001). This might be a plausible explanation for the lack of effect of NO_3_^−^ on MUP area as there is no existing data to our knowledge to indicate that NO_3_^−^ impacts recruitment of MUs. Accordingly, further research is required to assess the translational potential of the lower MUP duration after NO_3_^−^ supplementation to improve muscle contractility and the potential mechanism of this effect.

### Motor unit firing rate

MUFR decreased after 1 min and remained low for the rest of the ischemic task, consistent with previous literature (Bigland-Ritchie et al. 1986; Garland et al. 1994; Adam and De Luca 2005). Although speculative, reduced MUFR concomitant with increased MUP area during an ischemic sustained effort might be due to; (1) a decrease in MUFR of the active MUs during consistent force, (2) MUs that have low firing rates are initially activated, or (3) recruitment of new MUs that have lower firing rates than the initially active MUs (Bigland-Ritchie et al. 1986; Garland et al. 1994; Yasuda et al. 2006; Calder et al. 2008; McManus et al. 2015). The reduction in MUFR during the ischemic contraction may be linked to the accumulation of metabolites, such as inorganic phosphate, resulting in increased type III/IV afferent feedback and a subsequently inhibitory effect on central motor output (Amman et al. 2008, 2012; Rossman et al. 2012; Taylor et al. 2016). Although there is evidence that NO_3_^−^ supplementation can limit the increase of such metabolites (Bailey et al. 2010), MUFR was not different between the NIT and PLA conditions in the current study.

These findings conflict with the only previous study by Flanagan et al. (2016), that reported decreased MUFR during resistance exercise after NO_3_^−^ supplementation, despite a longer duration (5 vs. 3 days) and higher dose (~ 12.8 vs. 0.05 mmol/day) of NO_3_^−^ supplementation in our study. Since Flanagan et al. (2016) administered a sport bar that provided a small NO_3_^−^ dose, it is possible that other nutrients in the bar may have contributed to the effects observed. These disparate findings might be also related to differences in the skeletal muscle contractile tasks (dynamic exercise *vs.* isometric contractions), given that alterations in MUFR patterns are task-dependent (Enoka and Stuart 1992). Moreover, Flanagan et al. (2016) used sEMG to assess MUFR, whereas iEMG was used in the current study and this methodological difference might be another reason for this interstudy disparity. However, we cannot exclude the possibility that NO_3_^−^ supplementation could affect fatigue-induced alterations in MUFR, where the contractile task is performed without BFR, at higher (> 25%) submaximal or maximal forces, in different muscle groups, or during a different contractile task to that employed in the current study. Finally, since we used voluntary contractions, we cannot exclude the possibility that some effects could be due to altered central motor output. However, NO_3_^−^ supplementation does not appear to alter voluntary activation (Husmann et al. 2019); therefore, the effects could be more local to the muscle tissue.

### NF jiggle

To the best of our knowledge, this is the first study to reveal instability of neuromuscular transmissions during a 3 min isometric contraction performed with BFR. The increased NF jiggle during a sustained isometric ischemic contraction may have a negative impact on muscle contractile function, since higher NF jiggle indicates more transmission and firing variability from unstable NMJ which also occurs in the skeletal muscle with age (Hourigan et al. 2015; Piasecki et al. 2106a, b) and with increased contraction intensity (10 vs. 25%) (Guo et al. 2022).However, there was no effect of NO_3_^−^ supplementation on NF jiggle during isometric contractions or during a sustained isometric contraction performed with BFR in the current study.

### Limitations

Although the iEMG technique used in this study may provide an advantage with regards to sensitivity, sampling MUs only at a single contraction intensity can be considered as a limitation of this study. Given both MU activity and the efficacy of NO_3_^−^ supplementation to improve muscle contraction are considered to be task-dependent (Enoka and Stuart 1992; Jones et al. 2018), different intensities (e.g., higher) or/and different exercise tasks (e.g., intermittent) might impact the effect of NO_3_^−^ supplementation on MU activity. All female athletes who participated in this study were actively using hormonal contraceptives, which maintain female sex hormones at relatively constant levels throughout the menstrual cycle (Cicinelli et al. 1996), which would minimise any impact of natural fluctuations in these hormones on skeletal muscle contractility (Sarwar et al. 1996). However, it is acknowledged that a limitation of the present study is that we did not compare hormone concentrations within the females between conditions.

## Conclusions

These findings provide insight into the effect of NO_3_^−^ supplementation on MUP properties and reveal lower MUP duration during brief isometric contractions and a sustained ischemic muscle contraction after short-term NO_3_^−^ supplementation. These observations improve understanding of the effect of NO_3_^−^ supplementation on neuromuscular function in healthy adults and may have implications for enhancing skeletal muscle contractile function.

## Data Availability

Raw data are available upon request.

## Abbreviations

BFR: Blood flow restriction
Ca^2^^+^: Calcium
cGMP: Cyclic guanosine monophosphate
Cys: Cysteine
iEMG: Intramuscular electromyography
K^+^: Potassium
MU: Motor unit
MUFR: Motor unit firing rate
MUP: Motor unit potential
MUPT: Motor unit potential train
MVC: Maximum voluntary contraction
NF: Near fibre
NIT: Nitrate trial
NMJ: Neuromuscular junction
NO: Nitric oxide
NO_2_^−^: Nitrite
NO_3_^−^: Nitrate
PLA: Placebo trial
O_2_: Oxygen
sEMG: Surface electromyography
sGC: Soluble guanylyl cyclase
VL: Vastus lateralis
